# Superior Mesenteric Artery Thrombosis in a Patient With Median Arcuate Ligament Syndrome

**DOI:** 10.7759/cureus.39351

**Published:** 2023-05-22

**Authors:** Nicolette Cassim, Jason Diljohn, Fidel S Rampersad, Adrian Chan

**Affiliations:** 1 Radiology, University of the West Indies, St. Augustine, TTO

**Keywords:** mals, doppler ultrasound, abdominal ct angiography, ct angiography, gastro-intestinal symptoms

## Abstract

Median arcuate ligament syndrome (MALS) is a rare and controversial vascular compression syndrome. In this condition, the median arcuate ligament compresses the celiac artery, resulting in symptoms such as postprandial abdominal pain, vomiting, and weight loss. Its diagnosis is based on clinical findings in conjunction with supporting radiological features such as elevated flow velocities on Doppler ultrasound and focal indentation of the proximal celiac artery with the typical 'hooked' or 'J'-shaped appearance on conventional angiography or computed tomography angiography (CTA).

Herein is the case of a 44-year-old female who presented with early satiety, postprandial abdominal pain, vomiting, and weight loss. A computed tomography mesenteric angiogram (CTMA) showed thickening of the median arcuate ligament with a hooked appearance of the celiac artery and thrombosis of the mid to distal superior mesenteric artery with associated ischemia of a short segment of the jejunum. Subsequent Doppler ultrasound demonstrated elevated peak systolic velocities within the celiac artery over the compressed segment, which varied with respiration (end-inspiration: 234.3 cm/s and end-expiration: 373.5 cm/s).

## Introduction

Median arcuate ligament syndrome (MALS) is a rare and controversial entity in which the fibrous arch connecting the diaphragmatic crura compresses the celiac artery [1]. This compression results in hemodynamically significant stenosis of the celiac artery, which in turn causes symptoms such as postprandial abdominal pain, vomiting, and weight loss. It typically occurs in the younger age group (20-40 years) and is more common in females [2].

Computed tomography (CT) plays an important role in the evaluation of patients with suspected MALS as it is able to show thickening of the median arcuate ligament (>4mm), characteristic 'hooked' or 'J'-shaped appearance of the celiac artery, and collateral vessel formation [2]. Doppler ultrasound is also useful in demonstrating elevated peak systolic velocities, which vary during respiration (>200cm/s during expiration). Its diagnosis is one of exclusion and is based on a combination of clinical symptoms and radiological findings.

Herein we report a case of MALS in a patient with concomitant superior mesenteric artery (SMA) thrombosis.

## Case presentation

A 44-year-old thin female presented to the adult emergency department with a one-week history of postprandial epigastric pain and associated vomiting. A physical exam at this time was unremarkable. Routine blood investigations (complete blood count, liver function tests, and serum amylase) were normal. Abdominal ultrasound done at this excluded cholelithiasis and pancreatitis.

The patient returned one month later with vague/generalized abdominal pain. Blood investigations at this time showed an elevated white blood cell (WBC) count and elevated lactate dehydrogenase (LDH). Intravenous contrast-enhanced CT-mesenteric angiogram (MA) (done at the inspiratory phase of respiration) demonstrated SMA thrombus at its mid/distal segment with associated mild circumferential thickening and hyper-enhancement of a short segment of jejunum with surrounding fat stranding. The patient was referred to general surgery and internal medicine, who performed coagulation screens which were negative. The patient was also referred to vascular surgery based on these CT findings.

**Figure 1:**
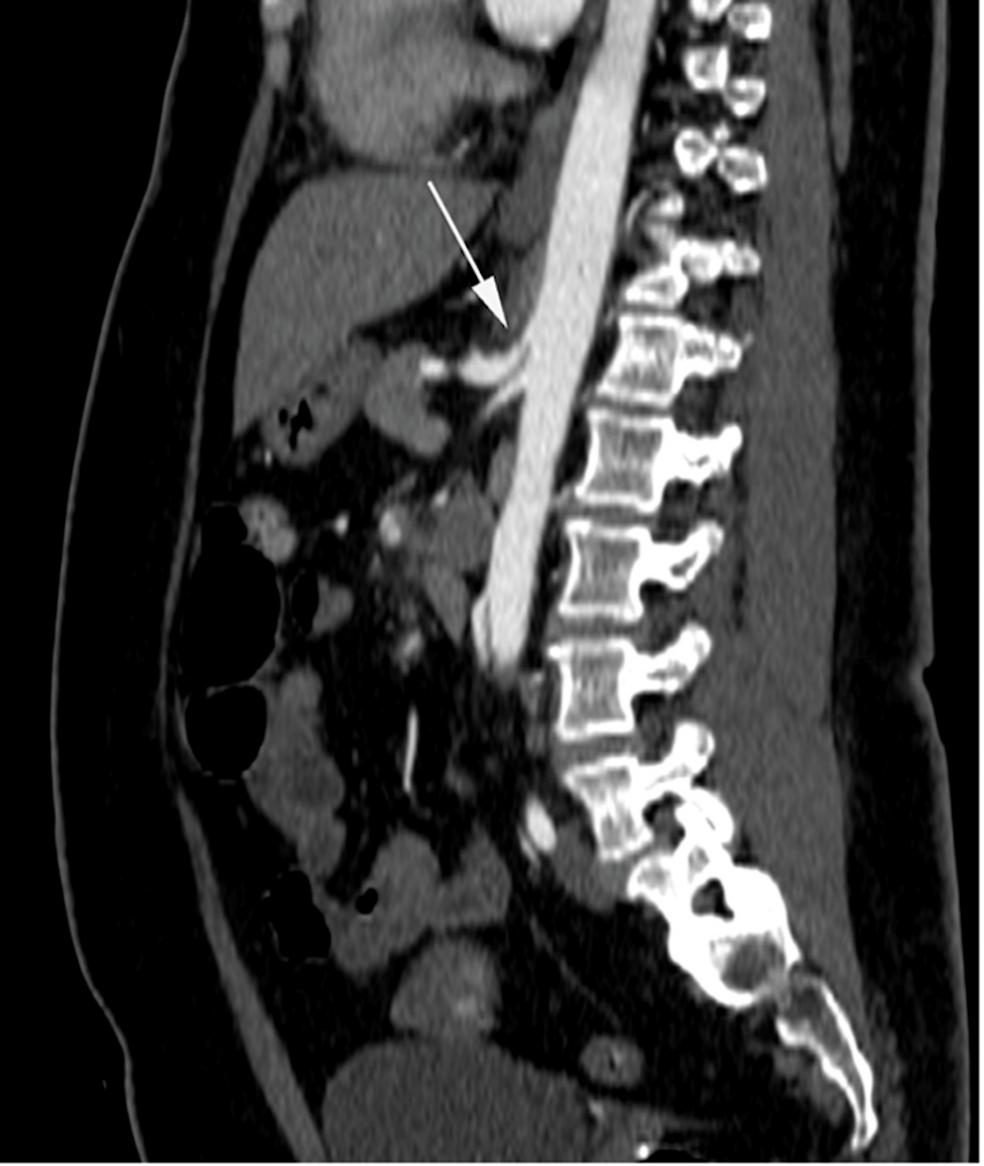
Sagittal reformatted, post intravenous contrast computed tomography White arrow demonstrates short segment stenosis of the coeliac trunk with a characteristic hooked appearance and post-stenotic dilatation.

**Figure 2:**
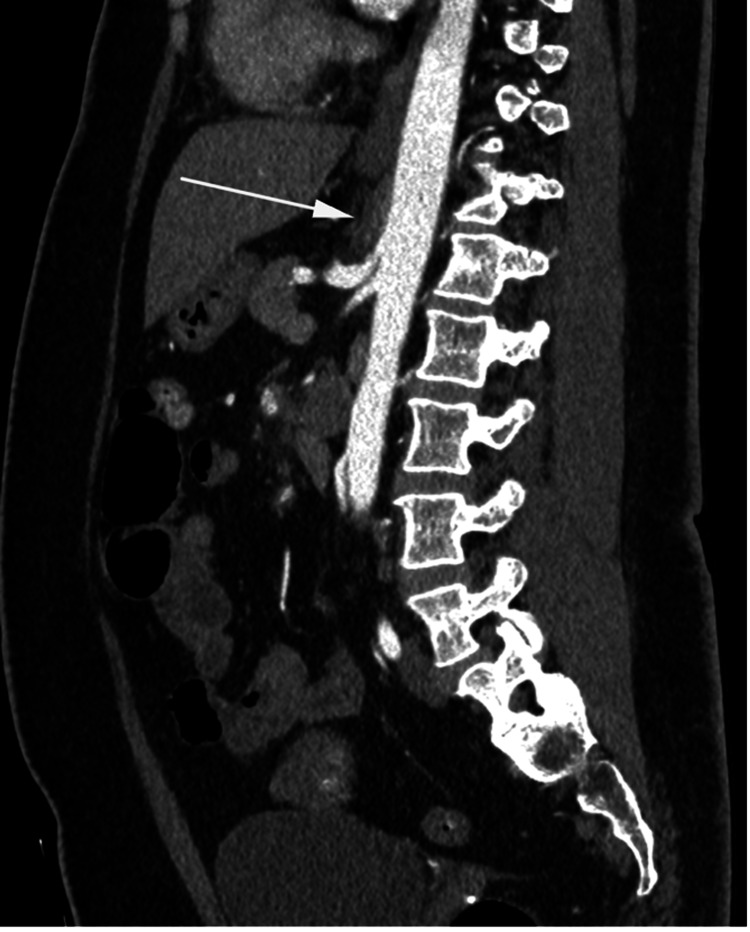
Sagittal reformatted, post intravenous contrast computed tomography White arrow demonstrates the thickening of the median arcuate ligament (MAL).

**Figure 3:**
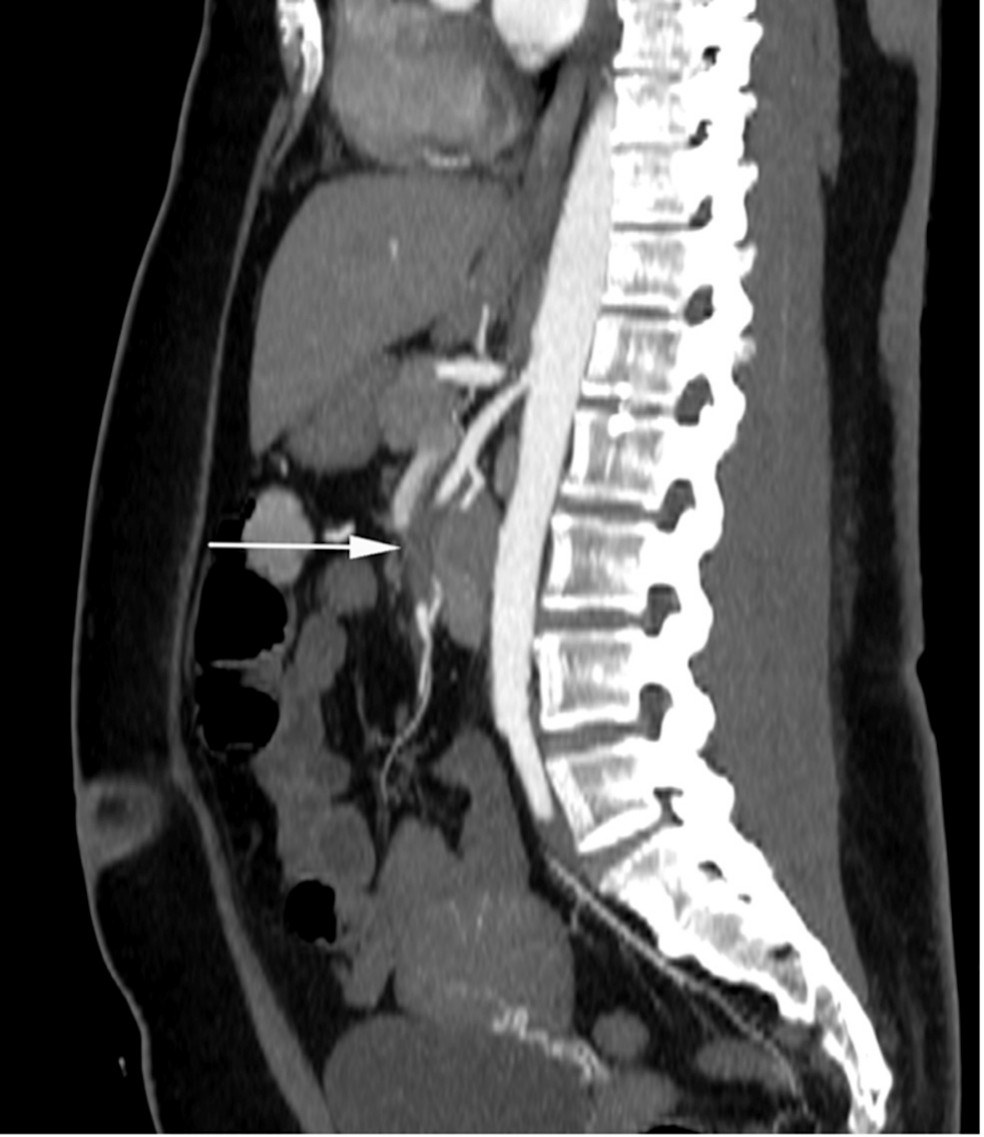
Sagittal reformatted, maximum intensity projection (MIP), post intravenous contrast computed tomography White arrow demonstrates occlusive thrombus in the mid/distal portion of the superior mesenteric artery (SMA).

**Figure 4:**
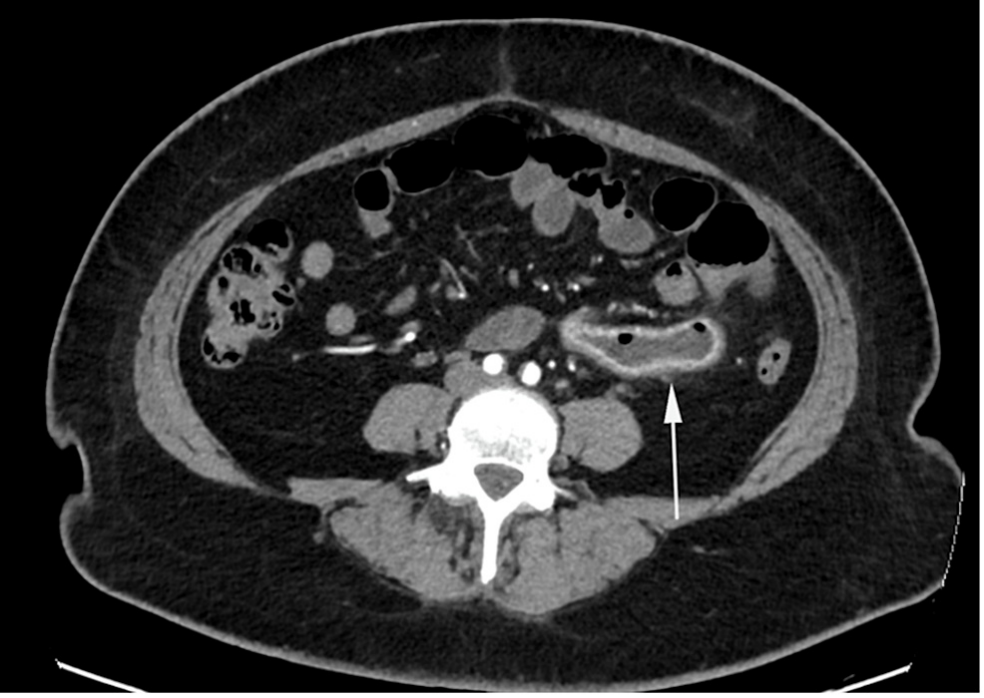
Axial reformatted, post intravenous contrast computed tomography images in the arterial phase White arrow demonstrates mild circumferential mural thickening and hyper-enhancement in a short segment of the jejunum.

Following correlation with clinical symptoms (early satiety, postprandial abdominal pain, vomiting, and weight loss) and signs (mid-abdominal bruit, which varied with respiration), a diagnosis of MALS was made. A review of the CTMA (Figures 1-3) also showed thickening of the median arcuate ligament (9 mm) and a hooked appearance of the celiac trunk with associated focal narrowing (3 mm) of its proximal portion and post-stenotic dilatation of its distal portion (8 mm). No atherosclerotic plaques were seen on CT. There was associated circumferential bowel wall thickening of a short segment of the jejunum (Figure 4). 

**Figure 5:**
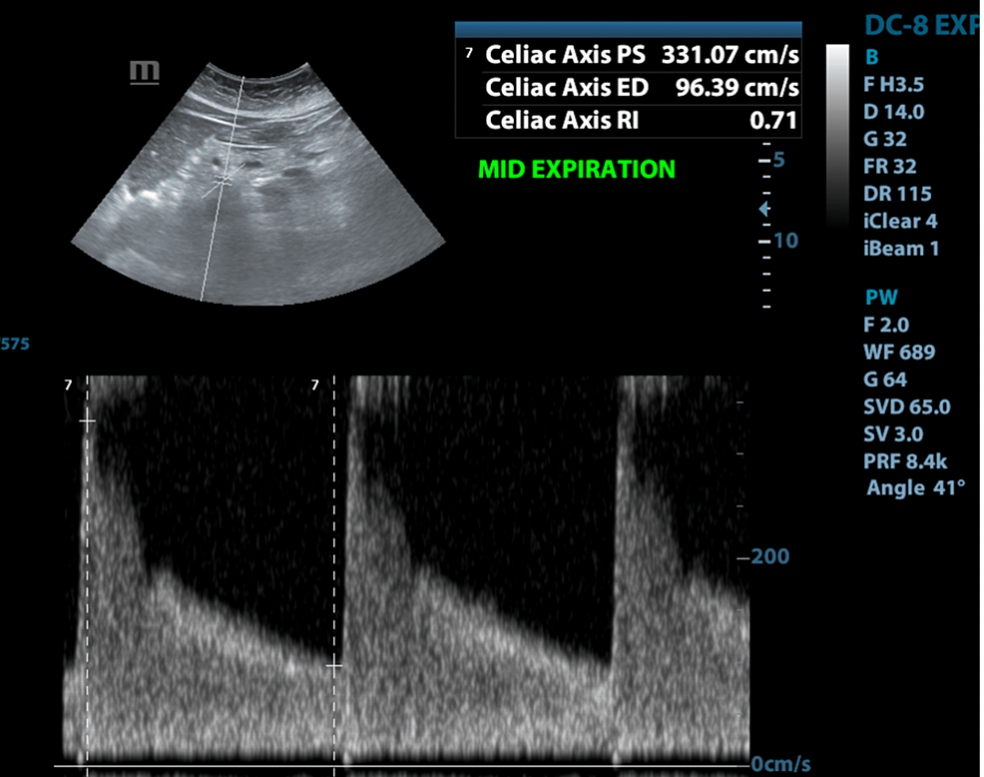
Spectral Doppler ultrasound in the mid-expiration over the compressed segment of the coeliac trunk demonstrating an elevated peak systolic velocity (PSV) of 331.07 cm/s

Spectral Doppler ultrasound (Figure 5) was subsequently performed and demonstrated elevated peak systolic velocities within the celiac artery over the compressed segment which varied with respiration (end-inspiration: 234.3 cm/s and end-expiration: 373.5 cm/s).

Open surgical division of the median arcuate ligament (MAL) was performed, and the ischemic segment of the jejunum was resected. Rivaroxaban was also started post-procedure for the SMA thrombus. There were no intra-procedural complications, and the postoperative period was uneventful, with early discharge. The patient indicated resolution of the symptoms on the follow-up visit.

## Discussion

MALS is a rare and controversial vascular compression syndrome. The median arcuate ligament is a ligamentous structure that connects the right and left diaphragmatic crura [3]. In 10-24% of the population, the MAL crosses over the proximal portion of the celiac artery due to either higher origin of the celiac artery or lower insertion of the MAL. In approximately 85% of individuals, this anatomical variation is asymptomatic [4]. However, compression of the celiac artery occurs in a small percentage of these individuals, with an even smaller subset having MALS (incidence 0.002-0.4%) [5,6,7]. It occurs most commonly in the 20-40 year age group and has a 71% female preponderance [8]. 

The typical clinical picture of MALS is one of a young, thin female with symptoms of nausea, vomiting, early satiety, postprandial epigastric pain (15-30 minutes after a meal), weight loss, and an abdominal bruit heard over the mid-epigastric region which varies with respiration [9]. These symptoms are non-specific and are often misdiagnosed as functional dyspepsia, peptic ulcer disease, gastropathy, or cholelithiasis [5].

There are two proposed theories on the cause of the symptoms of MALS: 1) mesenteric ischemia caused by compression of the celiac artery, and 2) direct irritation and compression of the celiac ganglion or plexus by the MAL [7]. 

Severe celiac artery stenosis may lead to altered hemodynamics in the SMA and collateral pathways, such as the pancreaticoduodenal arcade, with intimal damage and the formation of true aneurysms. SMA dissection in a patient with MALS has been described in one patient and was believed to be due to increased blood flow to the SMA in a hypertensive patient with possible underlying atherosclerosis [5,8]. In our patient, there was concomitant SMA thrombosis which may be due to altered hemodynamics in the SMA with resultant thrombus formation.

Previously, conventional angiography was considered the investigation of choice for MALS, allowing for dynamic assessment of the celiac artery with a demonstration of changes in compression during respiration [4,10]. During inspiration, the celiac artery assumes a more caudal orientation with the downward movement of the diaphragm. During expiration, compression of the celiac artery increases. True compression persists during end-inspiration, whereas transient compression may be seen only during end-expiration [10].

Conventional angiography has been largely replaced by CTA, which is performed at the end-inspiratory phase. This provides the added benefit of visualization of the median arcuate ligament (thickness of >4mm is considered abnormal), as well as atherosclerotic plaques and other causes of extrinsic compression. Focal indentation of the proximal celiac artery with the typical 'hooked' or 'J'-shaped appearance (best seen on sagittal CT images), post stenotic dilatation, and collateral pathways such as the pancreaticoduodenal arcade can be seen on both conventional and CT angiography [4,7]. 

Doppler ultrasound demonstrates dynamic variations of flow velocity in the celiac artery during respiration. A peak systolic velocity of greater than 200 cm/s has a reported sensitivity and specificity of 75% and 89%, respectively, in detecting stenosis of at least 70% [9]. Compression of the celiac artery is most severe at the end of expiration, with a corresponding marked increase in flow velocities [4,9]. The combination of a peak systolic velocity of greater than 350 cm/s at end-expiration and an angle of less than 50° between the celiac artery and aorta is a reliable indicator of MALS on ultrasound [11]. 

Surgical treatment is more likely to relieve symptoms in the 40-60 year age group, particularly in those with postprandial pain, greater than 20-lb weight loss, post stenotic dilatation, and those with collateral vessels [7,9]. The standard treatment of MALS involves surgical release of the celiac artery from compression via division of the MAL through either an open or laparoscopic approach. In some patients, the ligamentous construction causes vascular damage, which may require vascular reconstruction. Celiac ganglionectomy, aortoiliac bypass surgery, and reimplantation of the celiac artery may be performed in some patients. Intraoperative ultrasound may be used to confirm adequate release [4,12,13]. There is no reason to perform endovascular stenting of the celiac artery pre-operatively as these generally fail due to external compression by the MAL [13].

The above treatment options generally have good outcomes. The average success rate (symptom-free) after surgical intervention is 60-80%. Post-operative complications are generally minor and self-limited, including diarrhea, nausea, and pancreatitis. In those patients with persistent or recurrent abdominal pain, radiological re-evaluation for possible restenosis of the celiac artery with additional procedures such as angioplasty (for cases with elevated flow velocities on Doppler ultrasound) or celiac plexus nerve block (for cases with normal flow velocities on Doppler ultrasound and normal CT angiogram studies) [13].

## Conclusions

Median arcuate ligament syndrome is a rare and controversial vascular compression syndrome mainly occurring in females between the ages of 20-40 years. A combination of clinical and radiological signs is essential for diagnosis. Surgical treatment is usually done to relieve symptoms and involves surgical release of the celiac artery from compression via division of the MAL through either an open or laparoscopic approach. Celiac ganglionectomy, aortoiliac bypass surgery, and reimplantation of the celiac artery may also be performed. Surgical outcomes are usually good, with 60-80% of patients symptom-free post-procedure.
